# Effectiveness of Subcutaneous Tumor Necrosis Factor Inhibitors in Patients With Ankylosing Spondylitis: A Real-World Prospective Observational Cohort Study in China

**DOI:** 10.3389/fphar.2019.01476

**Published:** 2019-12-17

**Authors:** Xiaojian Ji, Yiwen Wang, Zhengyuan Hu, Yingpei Ma, Siliang Man, Kunpeng Li, Yanyan Wang, Jian Zhu, Jianglin Zhang, Feng Huang

**Affiliations:** ^1^Department of Rheumatology, Chinese PLA General Hospital, Beijing, China; ^2^State Key Laboratory of Kidney Disease, Chinese PLA General Hospital, Beijing, China

**Keywords:** tumor necrosis factor, ankylosing spondylitis disease activity, real-world study, adalimumab, biosimilar etanercept

## Abstract

**Objective:** This prospective observational study investigated the efficacy of tumor necrosis factor inhibitors (TNFis) on disease activity, physical functionality, and mobility in patients with ankylosing spondylitis (AS) in a real-world setting.

**Methods:** The Chinese Ankylosing Spondylitis Prospective Imaging Cohort (CASPIC) is an ongoing cohort study. Patients with AS were included to one of two groups: the TNFi user group included those who received TNFi at any time point; the non-TNFi user group included those who did not receive TNFi. Disease activity, physical functionality, and mobility were assessed by AS Disease Activity Scores (ASDAS), Bath AS Functional Index (BASFI), and Bath AS Metrology Index (BASMI), respectively.

**Results:** A total of 804 patients with AS (241 TNFi users and 563 non-TNFi users) were recruited. For TNFi users, 83% received an etanercept biosimilar and 17.0% received adalimumab. Seventy-three TNFi users (30.3%) discontinued TNFis during the follow-up period; the mean duration of TNFi treatment was 6.9 ± 3.2 months. Reductions in ASDAS were significantly greater in TNFi users than in nonusers at 3, 6, and 12 months (differences in ASDAS reduction were 0.61, 0.56, and 0.46 units, respectively, all *P* < 0.05). Similarly, the improvement in BASFI was significantly greater in users than in nonusers at 3, 6, and 12 months (differences in BASFI improvement: 0.31, 0.75, and 0.74 units, respectively, all *P* < 0.05). BASMI increased in nonusers at 6 and 12 months (0.27, *P* = 0.47; 0.66, *P* < 0.001, respectively), but did not change in users (−0.16 and −0.13, respectively, both *P* > 0.05). At 12 months, changes in BASMI were significantly greater in nonusers than in users (−0.60, *P* = 0.47).

**Conclusion:** TNFis are effective against disease activity and improve the physical functionality of patients with AS, even in those who taper or discontinue TNFis. Thus, TNFis may retard the progression of spinal mobility dysfunction in AS patients. TNF may maintain spinal mobility as indicated by the BASMI.

## Introduction

The most common form of spondyloarthritis is ankylosing spondylitis (AS), a chronic and progressive condition characterized by radiographic changes in sacroiliac joints (Braun and Sieper, 2007; Machado et al., 2010). Inflammation in AS mainly affects the axial skeleton, resulting in inflammatory back pain, bony fusion, and new bone formation in the spine. The peripheral joints, eyes, bowels, and lungs can also be affected by AS. In addition, AS, which has a global prevalence of 0.1–1.4%, (Dean et al., 2014) more commonly develops in young men.

Several randomized controlled trials have shown that tumor necrosis factor inhibitors (TNFis) improve the treatment of AS and spondyloarthritis (Calin et al., 2004; van der Heijde et al., 2005; van der Heijde et al., 2006; van den Berg et al., 2011; Ward et al., 2016; van der Heijde et al., 2017). In many randomized controlled trials, TNFis rapidly and significantly reduced disease activity, and their long-term use even delayed radiological progression of the spine (van der Heijde et al., 2015; Poddubnyy et al., 2016; Wei et al., 2018). However, in clinical settings, TNFi treatment may be tapered or even discontinued for various reasons, especially when TNFis are not fully covered by local healthcare services, such as in some developing countries like China (Glintborg et al., 2010; Kristensen et al., 2010).

The cost-effectiveness of TNFi therapy is an important factor in treatment decisions for both patients and rheumatologists (Braun et al., 2006). Although TNFi costs vary tremendously across countries, it is a financial burden worldwide for societies, families, and patients with AS (Westhovens and Annemans, 2016). In Western countries, the pressure to reduce medical cost has increased greatly (van der Heijde et al., 2017). In areas where TNFis are not fully covered by local healthcare services, including China and many other developing countries, this burden is higher and mainly borne by the patients.

In addition to cost considerations, long-term TNFi therapy is associated with increasing vulnerability to severe infectious diseases, including tuberculosis and hepatitis B infection, as well strains of carcinomas. Thus, in 2016, the Assessment of SpondyloArthritis International Society (ASAS)–European League Against Rheumatism (EULAR) recommended that tapering of TNFis should be considered in patients with sustained remission (at least 6 months) (van der Heijde et al., 2017). Because of the diversity of real-world settings and the inevitable existence of TNFi discontinuation or tapering, it is important to investigate the efficacy of TNFis in AS patients who had discontinued or tapered TNFi therapy. The primary purpose of this study was to model TNFi-related improvements in AS disease activity, physical functionality, and disability over a 12-month period. A secondary objective was to assess the effect of tapered TNFi therapy on outcomes in AS patients.

## Patients and Methods

### Patient Population and Inclusion Criteria

The Chinese Ankylosing Spondylitis Prospective Imaging Cohort (CASPIC) is a nationwide, ongoing, prospective, and state-funded cohort study which was launched in conjunction with Smart-phone SpondyloArthritis Management System, a mobile health (mHealth) (**Supplementary Figure 1**) (Ji et al., 2019). To observe the whole disease process, patients of any age and disease duration were enrolled, and their prognoses for AS were comprehensively evaluated. All data were obtained from the Chinese People’s Liberation Army (PLA) General Hospital, a prominent tertiary referral center in Beijing to which patients were referred from throughout the country. Patients were recruited consecutively from outpatient rheumatology clinics, irrespective of the presence of concomitant acute anterior uveitis, psoriasis, or inflammatory bowel disease. Eligible individuals for this study were patients who 1) fulfilled the 1984 modified New York criteria (van der Linden et al., 1984), 2) had complete clinical data including medical history, and 3) had at least one follow-up visit after the initial visit between April 2016 and April 2018. Exclusion criteria were patients who 1) refused to complete the survey and 2) had invalid/missing data on registration.

### Drug Exposure

TNFi users were defined as patients who were treated with TNFi at any point during the first and follow-up visits; the baseline was defined as the time of enrollment for patients who were using TNFi at the time of enrollment or as the time at which patients started using TNFi after enrollment. TNFi users received subcutaneous administration of TNFis, including biosimilar etanercept (ETN; Yisaipu^®^; Sunshine Guojian Pharmaceutical Co., Ltd.; Shanghai, China) or adalimumab (ADA; AbbVie, Ludwigshafen, Germany), a recombinant human tumor necrosis factor receptor–antibody fusion protein that is widely used in China (Huang et al., 2010; Huang et al., 2011; Li et al., 2018). Non-TNFi users were defined as patients who did not receive treatment with any type of TNFi during the observation period, and their baseline was defined as the time of enrollment in the registry. Patients who had at least one visit during the follow-up after the baseline were included for further analysis.

The use of other medications such as nonsteroidal anti-inflammatory drugs (NSAIDs) and conventional synthetic disease-modifying antirheumatic drugs (csDMARDs) such as methotrexate (Sine, Shanghai, China), sulfasalazine (Sine, Shanghai, China), and thalidomide (Changzhou Pharmaceutical Factory Co., Ltd., Changzhou, China), was also analyzed.

### Outcomes

The Ankylosing Spondylitis Disease Activity Score (ASDAS), a good indicator of disease activity, was used to evaluate the outcomes of AS patients (Aydin et al., 2010). It was calculated using a formula defined for assessing disease activity in AS patients (Garrett et al., 1994; Lukas et al., 2009; van der Heijde et al., 2009). The Bath Ankylosing Spondylitis Functional Index (BASFI), which includes 10 questions on 0–10 numeric rating scales) (Garrett et al., 1994), was used to assess patients’ daily life functions. The questionnaire was completed by patients at each clinic visit. The Bath Ankylosing Spondylitis Metrology Index (BASMI) (Jenkinson et al., 1994) was used to assess the mobility of the spine and hips of AS patients and was determined by trained rheumatologists at each clinic visit. The primary outcome was the improvement in ASDAS during the follow-up period. The secondary outcomes were changes in BASFI and BASMI values.

The demographic characteristics of patients, including age, sex, height, weight, smoking status, comorbidities, past medical history, onset date for back pain, human leukocyte antigen (HLA)-B27 status, presence of AS features (acute anterior uveitis, psoriasis, and colonoscopy- and pathology-confirmed diagnoses of inflammatory bowel disease), family history, enthesitis, and peripheral arthritis, were recorded. The follow-up assessment for AS included examination of inﬂammatory markers such as erythrocyte sedimentation rate and C-reactive protein. When C-reactive protein levels were below the limit of detection, a constant value of 2 mg/l was used to calculate ASDAS (Machado et al., 2015).

Subsequent visits were scheduled according to the patients’ needs (1–12 months) and were determined at each visit. Discontinuation of TNFi treatment was defined as a ≥45-day gap without TNFi treatment. Safety was evaluated at every visit, including the monitoring of adverse events and assessment of clinical laboratory results. The reasons for TNFi discontinuation were also recorded, including shared patient–physician decision after remission, patient decision without the physician’s guidance, adverse effects, lack of efficacy following sufficient dosage for more than 1 month, and other reasons, such as pregnancy plans and surgery.

### Dose Tapering Strategy

Treatment adherence was defined as the number of days of continuous use of TNFis. Tapering of TNFis was considered for cases in which disease activity was stable (ASDAS < 1.3) after 3 months on the standard dose regimen. Each patient’s preference was fully considered in the tapering strategy. The dosage was reduced in a step-by-step and patient-tailored manner, starting at 10-day intervals for ETN and 20-day intervals for ADA; intervals generally did not exceed 1 month.

### Statistical Analysis

All analyses were performed using Empower (R) (www.empowerstats.com, X&Y Solutions, Inc., Boston, MA) and R (http://www.R-project.org). Quantitative data are presented as means and standard deviation (SD). Student’s *t* test or one-way analysis of variance was used to identify significant differences in quantitative variables. Categorical data are presented as percentage (%). Chi-square tests were used to identify significant differences in categorical data.

General additive mixed models with smooth curve fitting are optimal for analyzing repeated measurements (Lin and Zhang, 1999). General additive mixed models were used to assess the relationship between follow-up duration (independent variable) and ASDAS, BASFI, and BASMI (dependent variables), stratified by TNFi treatment. Intercept and time were included as random terms. In these models, ASDAS, BASFI, and BASMI were assessed at the baseline visit and during all follow-up visits. All models used the same set of fixed effects that have been widely used in studies of TNFi and AS disease outcomes (Molnar et al., 2018). The following variables were measured or calculated at the baseline visit and entered into adjusted models as fixed effects: gender, disease duration, body mass index, HLA-B27 status, smoking status (self-reported as never or former/current), peripheral arthritis, and treatment with NSAIDs (user or nonuser) and csDMARDs (user or nonuser). The interaction terms between follow-up time and TNFi treatment were also evaluated.

General additive mixed models were also used to assess the relationship between follow-up duration and ASDAS among TNFi users stratified by enthesitis. A two-tailed *P* value <0.05 was considered statistically significant.

## Results

### Study Population

**Figure 1:**
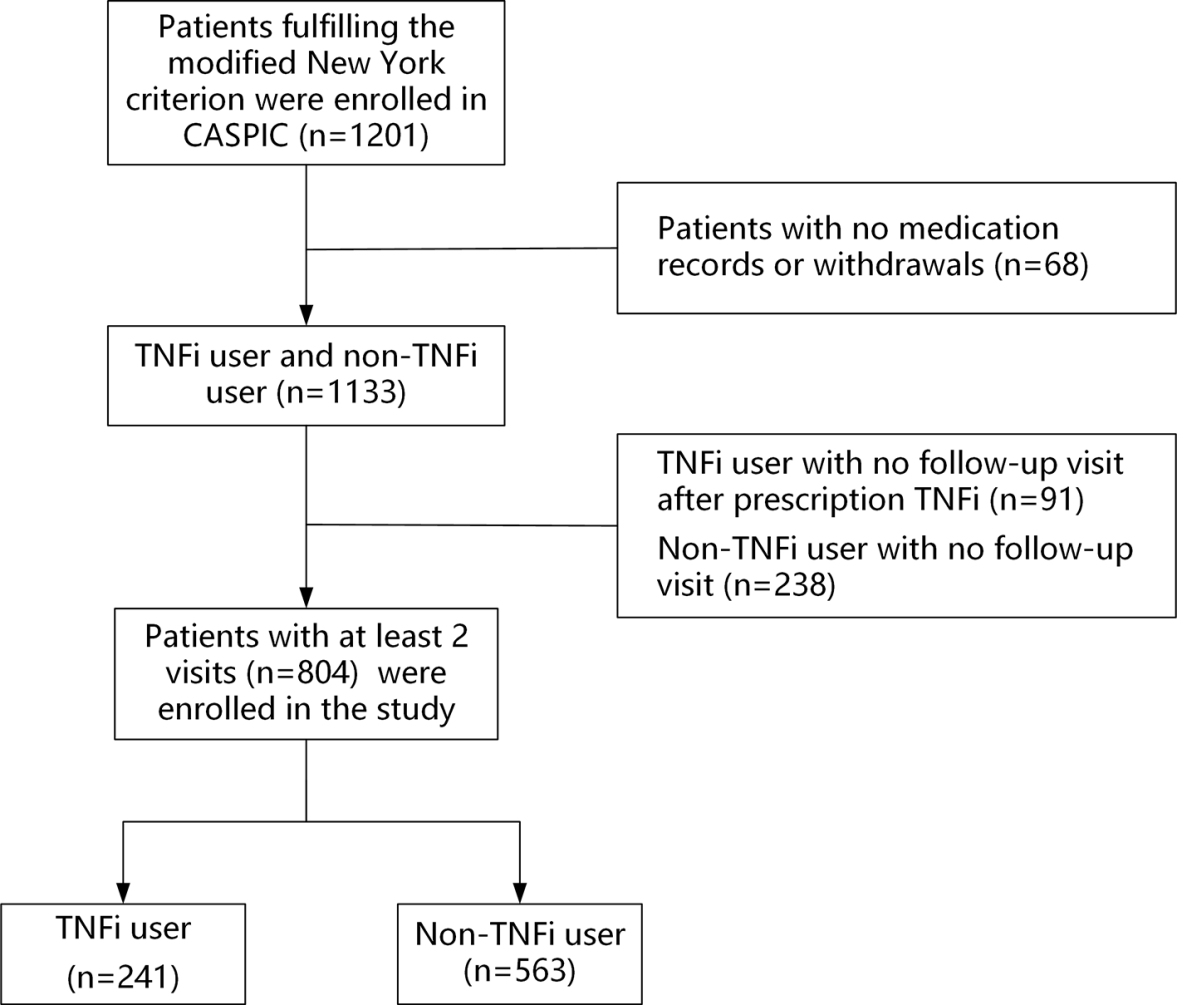
Participant selection flowchart. *CASPIC*, Chinese Ankylosing Spondylitis Prospective Imaging Cohort; *TNFi*, tumor necrosis factor inhibitor.

A total of 1,201 patients with AS were recruited between April 2016 and April 2018. Sixty-eight patients (5.7%) withdrew or had no medication records; 329 patients had no follow-up visits, including 91 (7.6%) TNFi users and 238 (19.8%) non-TNFi users. Those patients were excluded from the study (**Figure 1**). Finally, 804 patients with at least two follow-up visits were included in the study, including 241 TNFi users and 563 non-TNFi users. The mean patient age was 30.5 ± 8.8 years, and the majority of the patients were male (83.1%). The HLA-B27-positive rate was 88.7%. The median follow-up duration was 7.9 months (interquartile range, 0.9–12.0 months) in the TNFi user group and 7.5 months (interquartile range, 0.7–12.0 months) in the non-TNFi user group (*P* = 0.228). In the TNFi user group, 200 (83%) patients were given an ETN biosimilar and 41 (17.0%) patients were treated with ADA. The mean duration of TNFi treatment was 6.9 ± 3.2 months.

### Baseline Characteristics

The demographic characteristics of the 241 (30.0%) TNFi users and 563 (70.0%) nonusers are summarized in **Table 1**. The mean age was 29.0 ± 7.8 years for TNFi users and 31.1 ± 9.1 years for nonusers (*P* = 0.002). Compared with TNFi nonusers, TNFi users were younger and more likely to present with enthesitis and peripheral arthritis. In addition, TNFi nonusers had higher baseline disease activity scores (ASDAS), higher acute-phase reactant levels (higher erythrocyte sedimentation rate), more inflammation (higher C-reactive protein), and were less likely to receive NSAIDs and csDMARDs (all *P* < 0.05) than TNFi users. At baseline, ASDAS, BASFI, and BASMI scores significantly differed between non-TNFi users (2.0 ± 1.0, 1.5 ± 1.7, and 1.4 ± 2.0, respectively) and TNFi users (2.4 ± 1.1, 1.9 ± 1.8, and 2.0 ± 2.2, respectively; *P* < 0.05).

**Table 1:** Baseline characteristics of AS patients who were users and nonusers of tumor necrosis factor inhibitors.

Characteristics mean (SD) or %	Non-TNFi users (*n* = 563)	TNFi users (*n* = 241)	*P*
Male sex	83.8%	81.3%	0.385
Age, years	31.1 (9.1)	29.0 (7.8)	0.002
Disease duration, years	8.2 (6.5)	8.4 (5.6)	0.692
BMI, kg/m^2^	23.7 (3.9)	23.5 (3.9)	0.452
HLA-B27 positive	87.5%	91.5%	0.113
Smoker	32.4%	30.1%	0.521
BASFI	1.5 (1.7)	1.9 (1.8)	0.003
BASMI	1.4 (2.0)	2.0 (2.2)	0.006
ESR, mm/h	13.9 (15.8)	24.2 (24.7)	<0.001
CRP, mg/l	11.8 (25.1)	21.5 (28.1)	<0.001
ASDAS	2.0 (1.0)	2.4 (1.1)	<0.001
AAU	19.5%	23.8%	0.169
IBD	8.2%	12.6%	0.058
Psoriasis	3.9%	4.6%	0.668
Enthesitis	19.2%	32.4%	<0.001
Peripheral arthritis	9.2%	24.0%	<0.001
NSAID	98.9%	94.2%	<0.001
csDMARDs	71.8%	51.9%	<0.001

TNFi, tumor necrosis factor inhibitor; SD, standard deviation; BMI, body mass index; HLA-B27, human leukocyte antigen B27; BASFI, Bath Ankylosing Spondylitis Functional Index; BASMI, Bath Ankylosing Spondylitis Metrology Index; ESR, erythrocyte sedimentation rate; CRP, C-reactive protein; ASDAS, Ankylosing Spondylitis Disease Activity Score; AAU, acute anterior uveitis; IBD, inflammatory bowel disease; NSAID, nonsteroidal anti-inflammatory drug; csDMARDs, conventional synthetic disease-modifying antirheumatic drugs.

### Combination Therapy

**Table 2** summarizes the medications used by AS patients at baseline. NSAIDs were administered to 97.2% of patients and csDMARDs were administered to 64.8% patients; the csDMARDs included sulfasalazine (22.6% of patients), leflunomide (15.4% of patients), methotrexate (4.1% of patients), and thalidomide (23.5% of patients). TNFis were administered to 35.4% of patients. NSAID monotherapy and TNFi monotherapy were used in 18.0% and 1.3% of patients, respectively. NSAID plus csDMARD (46.2%) was the most common therapeutic regimen, followed by NSAID plus TNFi (15.8%). A combination regimen with three drugs was administered to 17.7% of patients. NSAIDs and thalidomide were less likely to be administered to female patients, who were more likely to receive TNFis as a monotherapy (4.2%).

**Table 2:** Baseline medications of patients on combination therapies and monotherapies.

Characteristics (%)	Overall (*n* = 804)
NSAIDs	97.2
TNFi	35.4
csDMARDs	64.8
Sulfasalazine	22.6
Leflunomide	15.4
Methotrexate	4.1
Thalidomide	23.5
Drug combination	
NSAID monotherapy	18.0
TNFi monotherapy	1.3
NSAID + csDMARD	46.2
NSAID + TNFi	15.8
NSAID + TNFi + csDMARD	17.7

NSAID, nonsteroidal anti-inflammatory drug; TNFi, tumor necrosis factor inhibitor; csDMARD, conventional synthetic disease-modifying antirheumatic drug.

### Efficacy

**Figure 2:**
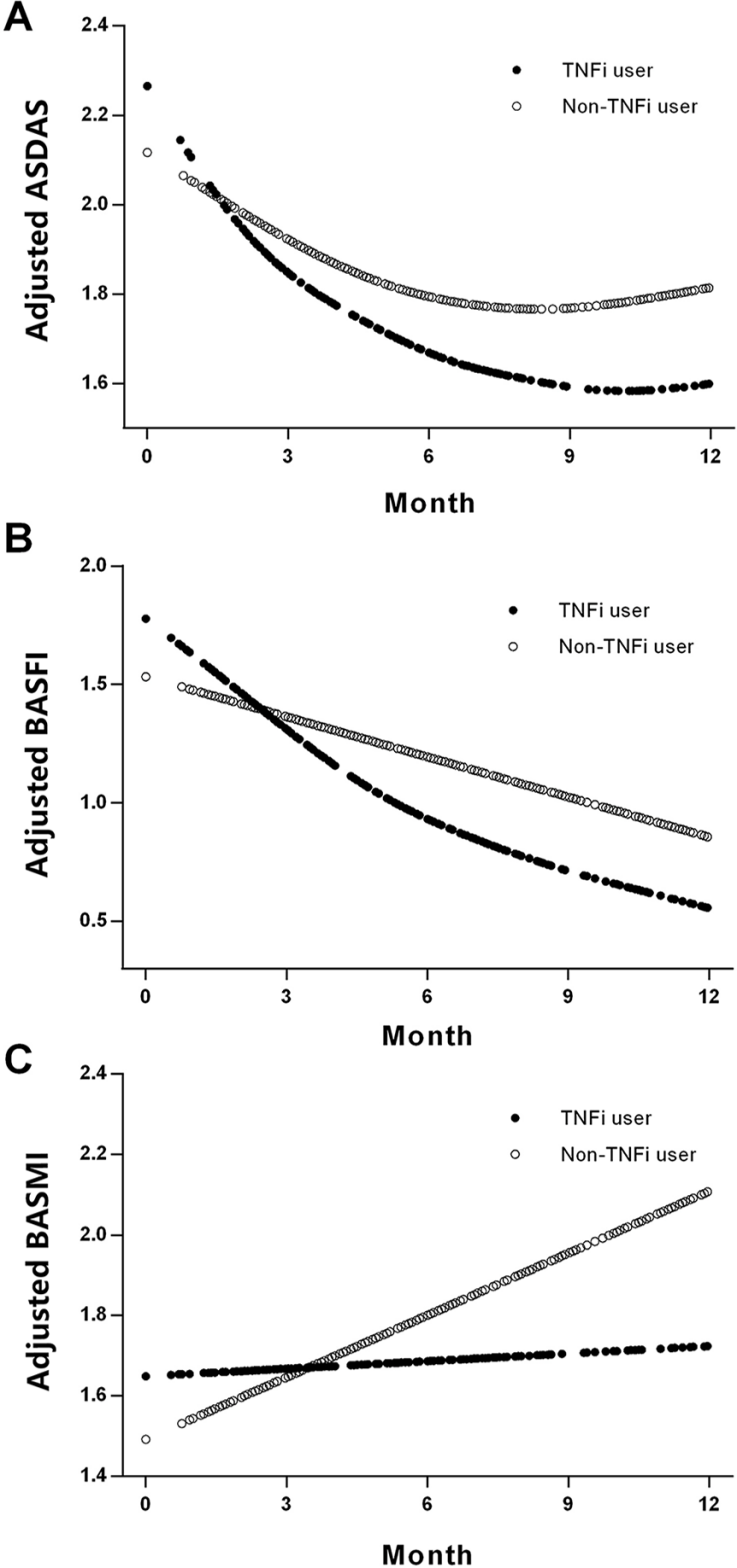
Smoothing analysis curves for ASDAS **(A)**, BASFI **(B)**, and BASMI **(C)** values during the follow-up of 12 months in TNFi and non-TNFi users. All of the models were adjusted for sex, symptom duration, human leukocyte antigen B27, body mass index, smoking status, peripheral arthritis, and treatment with nonsteroidal anti-inflammatory drugs and disease-modifying antirheumatic drugs. ASDAS, Ankylosing Spondylitis Disease Activity Score; BASFI, Bath Ankylosing Spondylitis Functional Index; BASMI, Bath Ankylosing Spondylitis Metrology Index; TNFi, tumor necrosis factor inhibitor.

**Figure 2** shows the smoothing curve analyses of ASDAS, BASFI, and BASMI values for TNFi users and non-TNFi users during the 12-month follow-up period. The adjusted decline in ASDAS among TNFi users and non-TNFi users over 3 months was 0.84 units [95% confidence interval (CI), 0.48–1.21; *P* < 0.001] and 0.21 units (95% CI, 0.015–0.58; *P* = 0.259), respectively (**Table 3**). There were significant differences in ASDAS decline between TNFi users and non-TNFi users during the 0- to 3-month, 0- to 6-month, and 0- to 12-month follow-up periods (0.61 units, *P* = 0.017; 0.56 units, *P* < 0.001; 0.46 units, *P* < 0.001, respectively).

**Table 3:** Unadjusted and adjusted changes in ASDAS, BASFI, and BASMI among TNFi users and nonusers.

	0–3 months		0–6 months		0–12 months	
	*β* (95% CI)	*P*	*β* (95% CI)	*P*	*β* (95% CI)	*P*
ASDAS						
Unadjusted						
Non-TNFi users	−0.38 (−0.66, −0.09)	0.012*	−0.49 (−0.66, −0.32)	0.018*	−0.41 (−0.54, −0.28)	<0.001*
TNFi users	−1.11 (−1.46, −0.77)	<0.001*	−0.90 (−1.16, −0.64)	<0.001*	−0.74 (−0.95, −0.52)	<0.001*
Difference	−0.80 (−1.24, −0.36)	<0.001*	−0.42 (−0.73, −0.12)	0.005*	−0.33 (−0.57, −0.09)	<0.001*
Adjusted						
Non-TNFi users	−0.21 (−0.58, 0.15)	0.259	−0.40 (−0.60, −0.21)	<0.001*	−0.44 (−0.63, −0.25)	<0.001*
TNFi users	−0.84 (−1.21, −0.48)	<0.001*	−0.94 (−1.22, −0.66)	<0.001*	−0.93 (−1.26, −0.41)	<0.001*
Difference	−0.61 (−1.13, −0.10)	0.023*	−0.56 (−0.89, −0.24)	<0.001*	−0.46 (−0.81, −0.10)	0.012*
BASFI						
Unadjusted						
Non-TNFi users	0.00 (−0.07, 0.07)	0.933	−0.56 (−0.74, −0.38)	<0.001*	−0.67 (−0.82, −0.52)	<0.001*
TNFi users	−0.22(−0.41, −0.03)	0.028*	−1.24 (−1.54, −0.94)	<0.001*	−1.25 (−1.52, −0.99)	<0.001*
Difference	−0.24(−0.45, −0.03)	0.024*	−0.69 (−1.02, −0.36)	<0.001*	−0.58 (−0.87, −0.29)	<0.001*
Adjusted						
Non-TNFi users	−0.02 (−2.62, 5.54)	0.482	−0.48 (−0.67, −0.29)	<0.001*	−0.67 (−0.92, −0.44)	<0.001*
TNFi users	−0.28 (−0.51, −0.05)	0.021*	−1.20 (−1.54, −0.86)	<0.001*	−1.45 (−1.87, −1.03)	<0.001*
Difference	−0.31 (−0.57, −0.05)	0.020*	−0.75 (−1.13, −0.38)	<0.001*	−0.74 (−1.18, −0.29)	0.001*
BASMI						
Unadjusted						
Non-TNFi users	0.12 (−0.38, 0.63)	0.630	0.23 (−0.09, 0.56)	0.158	0.68 (0.44, 0.93)	<0.001*
TNFi users	0.24 (−0.45, 0.94)	0.499	−0.06 (−0.64, 0.51)	0.833	0.06 (−0.50, 0.62)	0.840
Difference	0.15 (−0.68, 0.99)	0.718	−0.24 (−0.86, 0.38)	0.453	−0.53 (−1.08, 0.02)	0.060
Adjusted						
Non-TNFi users	0.10 (−0.34, 0.53)	0.670	0.27 (0.01, 0.53)	0.047*	0.66 (0.41, 0.91)	<0.001*
TNFi users	0.06 (−0.39, 0.50)	0.811	−0.16 (−0.82, 0.51)	0.639	−0.13 (−0.78, 0.51)	0.688
Difference	−0.11 (−0.74, 0.52)	0.729	−0.26 (−0.93, 0.40)	0.443	−0.60 (−1.19, −0.01)	0.047*

The model was adjusted for sex, symptom duration, human leukocyte antigen B27, body mass index, smoking status, peripheral arthritis, and treatment with non-steroidal anti-inflammatory drugs and disease-modifying antirheumatic drugs.

CI, confidence interval; ASDAS, Ankylosing Spondylitis Disease Activity Score; TNFi, tumor necrosis factor inhibitor; BASFI, Bath Ankylosing Spondylitis Functional Index; BASMI, Bath Ankylosing Spondylitis Metrology Index.

*P < 0.05.

For non-TNFi users, BASFI did not significantly decline during the first 3 months (0.02 units, *P* = 0.482), but they did significantly decline during the 0- to 6-month and 0- to 12-month follow-up periods (0.48 and 0.67 units, respectively, both *P* < 0.001). For TNFi users, BASFI significantly declined during the 0- to 3-month, 0- to 6-month, and 0- to 12-month follow-up periods (0.28 units, *P* = 0.021; 1.20 units, *P* < 0.001; 1.45 units, *P* < 0.001, respectively). The differences in BASFI reduction rates between TNFi users and non-TNFi users during each follow-up period were significant (**Table 3**).

For non-TNFi users, BASMI significantly increased during the 0- to 6-month and 0- to 12-month follow-up periods (0.27 units, *P* = 0.047, and 0.66 units, *P* < 0.001, respectively), whereas for TNFi users, the BASMI was stable—even slightly improved—during the same follow-up periods (0.06, −0.16, and −0.13 units, all *P* > 0.05). Differences in the reduction of BASMI between TNFi users and non-TNFi users were significant for the 12-month follow-up period (0.60 units, *P* = 0.047; **Table 3**).

The adjusted decline in ASDAS among TNFi users with and without enthesitis was 1.66 units (95% CI, 1.02–2.31; *P* < 0.001) and 0.60 units (95% CI, 0.22–0.99; *P* = 0.003), respectively, during the 12-month follow-up periods. Differences in the reduction of ASDAS between the two groups were significant for the 12-month follow-up period (1.13 units, *P* = 0.002; **Supplementary Table 1**). **Supplementary Figure 2**shows smoothing analysis curves for ASDAS during the follow-up of 12 months among patients with and without enthesitis among TNFi users.

### Discontinuation of TNFi

No other serious adverse events were observed during the follow-up period. TNFi-related adverse events occurred among six patients in the TNFi user group; therefore, TNFi was discontinued for those patients, including two patients with infections in the upper respiratory tract and one patient each with pulmonary tuberculosis, new-onset uveitis, palmoplantar pustulosis, and subacute thyroiditis. All of them were receiving adequate TNFi dosages (ADA: 40 mg/14 days; ETN biosimilars: 50 mg/7 days).

### TNFi-Related Adverse Events

No serious adverse events were observed during the follow-up period. TNFi-related adverse events occurred among six patients in the TNFi user group; therefore, TNFi was discontinued for these patients, including two patients with infections in the upper respiratory tract, one patient with pulmonary tuberculosis, one patient with new-onset uveitis, one patient with palmoplantar pustulosis, and one patient with subacute thyroiditis. All of them received adequate TNFi dosages (ADA: 40 mg/14 days; ETN biosimilars: 50 mg/7 days).

## Discussion

In this real-world cohort study of AS patients, those who received TNFi treatment showed better improvement in disease activity and physical functionality, and were more likely to maintain mobility, than those not treated with TNFis.

Clinical practice guidelines in 2016 recommended that tapering of TNFi therapy should be considered in patients with sustained remission (minimum of 6 months), owing to the high cost and risks of severe infectious disease associated with TNFi therapy (van der Heijde et al., 2017). Previous studies in Europe have reported that the first-year survival rate for AS patients with TNFi treatment is 75–88% (Pavelka et al., 2009; Lie et al., 2011; Heinonen et al., 2015). In countries where medical insurance does not cover the cost of TNFis, patients may not be able to afford the high cost of TNFi therapy for prolonged periods, and dose adjustments are important and pressing. In the present study, 30.3% of TNFi users discontinued TNFis by their final visit. At least 17.8% of those patients discontinued TNFis on their own for economic reasons. Economic factors also influenced shared patient–physician decisions to taper TNFi therapy after 3 months of full-dose treatment and to discontinue after 6 months if clinical improvement was achieved. Adverse events were also a reason for the discontinuation of TNFi therapy. Overall, the treatment with TNFi was well tolerated and only a few patients discontinued because of adverse events. In our study, six patients discontinued treatment due to adverse events, accounting for 8.2% of all discontinued patients treated with TNFi therapy. The proportion of discontinuation due to adverse events was similar to that reported of 8% (69/310) in the Danish nationwide DANBIO registry (Glintborg et al., 2010) and less than that reported of 27% in another observational study (Arends et al., 2011). Treatment and discontinuation strategies vary across countries. However, our results confirmed the real-world efficacy of TNFi for the treatment of AS, with respect to disease activity, physical functionality, and mobility. However, our results confirmed the real-world efficacy of TNFis for the treatment of AS, with regard to disease activity and physical functionality. Poddubnyy (Poddubnyy et al., 2016; Poddubnyy et al., 2018) reported that the functional status and spinal mobility of patients with established AS remained stable during long-term TNFi therapy during the observation period of 10 years. In our cohort, we also found that the BASMI of TNFi users was well maintained.

Although csDMARDs are not included in the ASAS-EULAR management recommendations (van der Heijde et al., 2017), rheumatologists have continued to use them in combination therapy according to national guidelines and their treatment experience in clinical practice. A Finnish observational study showed that 78% of patients with AS receive csDMARDs (Heinonen et al., 2015), whereas a Swedish study reported that 61% of patients with AS receive csDMARDs (Kristensen et al., 2010). In our study, csDMARDs were administered to 64.8% of patients, which is similar to those previous studies. TNFi users were less likely to use csDMARDs than non-TNFi users (51.9% and 71.8%, respectively, *P* < 0.001). Despite their lower rate of csDMARD use, TNFi users exhibited greater improvement in AS disease activity, physical functionality, and disability than non-TNFi users did during the 12-month follow-up period. More importantly, the decline in disease activity at the 3-month follow-up was four times greater for TNFi users than that for non-TNFi users. Therefore, the use of concomitant csDMARDs did not alter the efficacy of TNFis in this study.

Compared to male patients, female patients were much more likely to be prescribed monotherapy and had significantly lower NSAID and thalidomide intake rates. This may be because female patients have better spinal mobility and less severe structural damage than male patients do (van der Horst-Bruinsma et al., 2013; van der Slik et al., 2019). In addition, thalidomide has significant teratogenic toxicity and is not recommended for female patients of reproductive age.

Strengths of our study lie in the availability of data from an established observational cohort that included a continuous included sample of patients with AS, which may have aided in the reduction of biases in selection and observation. In addition, we used generalized additive mixed models to effectively analyze repeated measurements and individually aggregated data (Lin and Zhang, 1999). Despite the strengths of this study, there are still limitations to note. First, because it is a real-world, non-randomized comparative effectiveness study, the comparability of the TNFi and non-TNFi groups is potentially limited owing to confounding factors. Thus, to improve the comparability, we have adjusted several confounding factors including gender, disease duration, body mass index, HLA-B27 status, smoking status, peripheral arthritis, and treatment with NSAIDs and csDMARDs in the longitudinal model. Second, ADA and ETN were the only types of TNFis included in our study, and infliximab was not included because the number of patients using infliximab on an outpatient basis was very low. Other types of TNFis are unavailable in China. Third, the sample was included in a single tertiary academic center, which may limit the ability to extrapolate our ﬁndings to other clinical settings.

## Conclusions

In summary, we found that after adjustment for mixed factors, short-term treatment with TNFis was associated with significant improvements in disease activity and physical functionality in patients with AS, whereas therapies using only NSAIDs and csDMARDs (non-TNFi users) were significantly less effective than TNFis for improving disease activity, increasing physical functionality, and maintaining spinal mobility as indicated by the BASMI.

## Data Availability Statement

The data used to support the findings of this study are available from the corresponding author upon request.

## Ethics Statement

The studies involving human participants were reviewed and approved by The Ethics Committee of the Chinese PLA General Hospital (S2016-049-02). Written informed consent to participate in this study was provided by the participants’ legal guardian/next of kin.

## Author Contributions

Conception and design: FH. Administrative support: JZhu and JZha. Data analysis and interpretation: XJ. Data collection, manuscript writing and final approval of the manuscript: XJ, YiW, ZH, YM, SM, KL, YaW, JZhu, JZha and FH.

## Funding

This work was supported by the Key Projects in the National Science & Technology Pillar Program during the Twelfth Five-Year Plan Period (2014BAI07B05) and the National Key Basic Research Program of China (973 Program; 2014CB541806).

## Conflict of Interest

The authors declare that the research was conducted in the absence of any commercial or financial relationships that could be construed as a potential conflict of interest.
