# Supplementary material for: Effectiveness of Subcutaneous Tumor Necrosis Factor Inhibitors in Patients With Ankylosing Spondylitis: A Real-World Prospective Observational Cohort Study in China
Source: Front Pharmacol. 2019 Dec 17;10:1476. doi: 10.3389/fphar.2019.01476 (PMC6929657; doi:10.3389/fphar.2019.01476)
Supplement: Supplementary file 1 [file DataSheet_1.docx]

**Supplementary documents**

**Table 1** Adjusted changes in ASDAS among patients with and without enthesitis among TNFi users

|  | 0–12 months | |
| --- | --- | --- |
| Adjusted ASDAS | β (95% CI) | *P* |
| Without enthesitis | -0.60 (-0.99, -0.22) | 0.003* |
| With enthesitis | -1.66 (-2.31, -1.02) | < 0.001* |
| Difference | -1.13 (-1.82, -0.44) | 0.002* |

**Notes:** ^*^*P* < 0.05. The model was adjusted for sex, symptom duration, human leukocyte antigen B27, body mass index, smoking status, peripheral arthritis, and treatment with non-steroidal anti-inflammatory drugs and disease modifying antirheumatic drugs. Abbreviations: CI, confidence interval; ASDAS, Ankylosing Spondylitis Disease Activity Score; TNFi, tumor necrosis factor inhibitor.


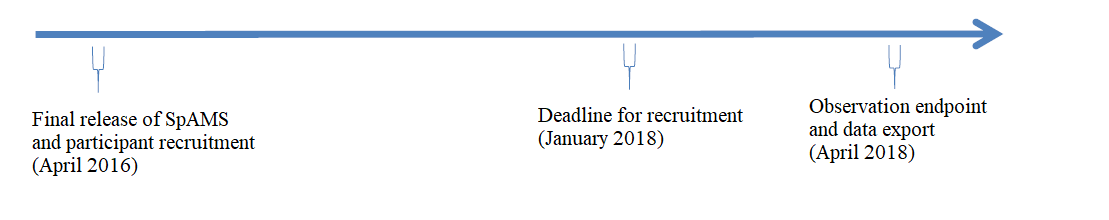
 **Figure 1.** Timeframes for data inclusion


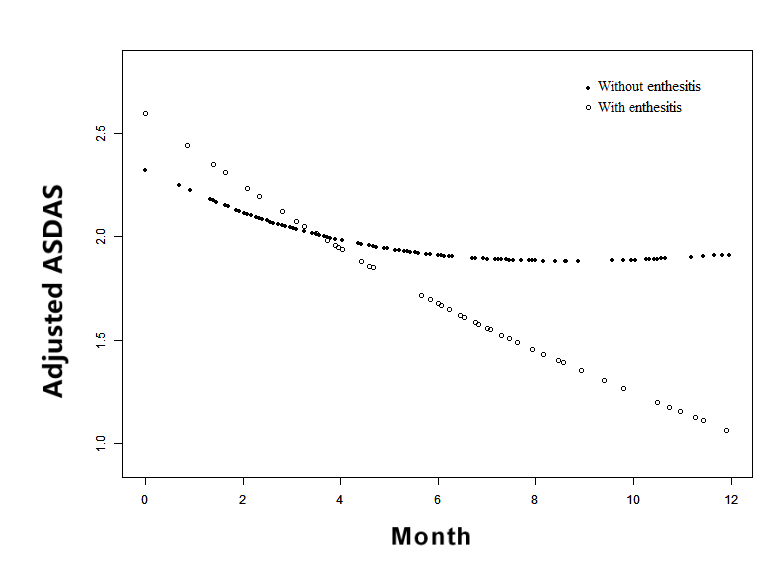


**Figure 2** Smoothing analysis curves for ASDAS during the follow-up of 12 months among patients with and without enthesitis among TNFi users. The model was adjusted for sex, symptom duration, human leukocyte antigen B27, body mass index, smoking status, peripheral arthritis, and treatment with non-steroidal anti-inflammatory drugs and disease modifying antirheumatic drugs. Abbreviations: ASDAS, Ankylosing Spondylitis Disease Activity Score.
